# PReS13-SPK-1212: Vaccination in paediatric patients with auto-immune and autoinflammatory diseases

**DOI:** 10.1186/1546-0096-11-S2-I30

**Published:** 2013-12-05

**Authors:** N Wulfraat

**Affiliations:** 1 Pediatrics, University Medical Center Utrecht, Utrecht, The Netherlands

## 

N.M. Wulffraat and MW Heijstek, Department of pediatric rheumatology, UMC Utrecht, The Netherlands

In order to develop evidence-based recommendations for vaccination of pediatric patients with auto-immune and/or auto-inflammatory diseases, a EULAR task force performed a systematic literature review. Available evidence was critically appraised using a customary scoring system for the level of evidence. The strength of each recommendation was determined.

The majority of papers considered influenza (44) or pneumococcal (20) vaccination. Very few studies were found for the live-attenuated vaccines. Considering composite vaccines, it is recommended to adhere to national guidelines for the meningococcal serogroup C conjugate, Hib, pneumococcal, hepatitis A and B, DTaP, HPV, Japanese encephalitis, tick-borne encephalitis, typhoid fever, rabies and cholera vaccination (Grade C-D). Seasonal influenza vaccination is recommended (Grade D). Patients on anti-CD20 therapy must receive tetanus specific immunoglobulines when indicated, since rituximab lowers responses to tetanus toxoid (Grade D).

Since the publication of these recommendations, we concluded a randomised controlled trial for the effects of booster MMR in 139 children (60 using MTX and 15 using a biological) with JIA in the age of 4 to 9 years. Disease activity measured by the JADAS 27 did not differ between the revaccinated and the non-vaccinated groups. As expected seroprotection rates were higher in the revaccinated group. Methotrexate and biologicals did not affect humoral responses, but low numbers precluded definite conclusions.

## Disclosure of interest

N. Wulfraat Consultant for: Pfizer, Novartis, Roche, AbbVie.

